# Non-specific alarm calls trigger mobbing behavior in Hainan gibbons (*Nomascus hainanus*)

**DOI:** 10.1038/srep34471

**Published:** 2016-09-30

**Authors:** Huaiqing Deng, Kai Gao, Jiang Zhou

**Affiliations:** 1 School of Life Sciences, Guizhou Normal University, Guiyang, Guizhou, 550001, China, 116# Baoshan North Road, Guiyang, Guizhou 550001, China

## Abstract

Alarm calls are important defensive behaviors. Here, we report the acoustic spectrum characteristics of alarm calls produced by Hainan gibbons (*Nomascus hainanus*) inhabiting Bawangling National Nature Reserve in Hainan, China. Analysis of call data collected from 2002–2014 shows that alarm calls are emitted by all family group members, except infants. Alarm behavior included simple short alarming calls (7–10 min) followed by longer variable-frequency mobbing calls lasting 5–12 min. The duration of individual alarming and mobbing calls was 0.078 ± 0.014 s and 0.154 ± 0.041 s at frequency ranges of 520–1000 Hz and 690–3920 Hz, respectively. Alarming call duration was positively associated with group size. The alarm calls can trigger mobbing behavior in Hainan gibbons; this is a defense way of social animals, and first report among the primates’ species. The system of vocal alarm behavior described in this critically endangered species is simple and effective.

Upon detecting a potential predator, many primates emit acoustic signals that influence the predator and cause group members to behave in a way that benefits the caller, regardless of relatedness or group association^1^. If the alarm call is emitted in a timely and reliable manner after predator detection, individuals in the group can respond and reduce their risk of being preyed upon^2^. Therefore, alarm calls are important defensive behaviors in animals and encode a rich amount of information^3^,^4^.

*Nomascus* spp. is small, territorial, social and arboreal apes distributed in Southeast Asia and Yunnan, Guangxi and Hainan provinces of China^5^. All adult gibbons make loud, continuous, complex and stable calls^6^. In the face of an external threat, gibbons also produce signals that elicit escape responses in conspecifics^7^. Predator-induced songs were identical to normal songs in the call note repertoire, for example, Lar gibbons (*Hylobates lar*) generate significant, context-dependent acoustic variations of their main social call, which potentially allows recipients to make inferences about events experienced by the caller^8^,^9^,^10^,^11^. Particular call types may be closely linked to function^12^.

We conducted field singing behavior research on critically-endangered Hainan gibbons (*Nomascus hainanus*) inhabiting Bawangling National Nature Reserve (BNNR) for 12 consecutive years from 2002 to 2013. We recorded singing behavior in three family groups and six solitary males, and have previously described the adult morning song, solo song and chorus in this species^13^. Here, we report alarm call behavior and acoustic spectrum characteristics and attempt to answer the following questions: Does the Hainan gibbon use different alarm calls for different predators? What is the composition and highest and lowest frequency of the alarm call? Is alarm call duration associated with group size? And what is the response time of individuals to alarm calls?

## Results

### Alarm call behavior

Alarm calls were emitted by family groups only and not solitary animals. Hainan gibbons produce two types of alarm call. In 15 alarm call records, seven were in response to humans, five to raptors and three to snakes. Hainan gibbons emitted the same alarm call across different threats (raptor, snake or people). Male and female adult gibbons emitted “gou-gou-gou” to alert group members of a danger when they encountered a potential predator, hereafter termed an alarming call. Following the alarming call, individuals in the family group gather, young gibbons move rapidly towards females, adult males conceal themselves in a location convenient for further vigilance, and all members (except infants) produce “jier-jier-jier” sounds, occasionally accompanied by jumping in a tree or flapping tree branches; we term the group call following the alarming call the mobbing call hereafter. During mobbing calls, gibbons occupied the crown canopy, remained relatively concealed while calling and continued to survey the external environment.

### Acoustic spectrum characteristics of alarming calls

**Figure 1:**
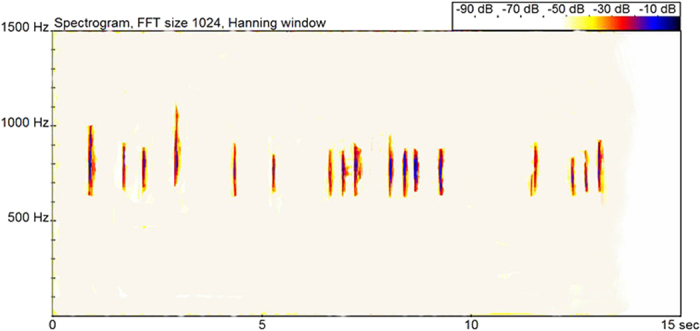
The alarming call in a group of Hainan gibbons (group B).

The alarming call was composed of short simple syllables (aa notes; Fig. 1). Different individuals in a group often repeated these simple syllables. The duration of an alarming call was 7–10 min (Table 1). There was a difference in the duration of alarming calls among the three groups (ANOVA: F = 8.42, df = 14, *P* < 0.05), and the duration was longer in larger groups (A > B > C). Acoustic spectrum analysis indicated that the mean duration of a syllable in an alarming call was 0.078 ± 0.014 s and 520–1000 Hz (Fig. 1, Table 2). Acoustic frequency differed between groups, and a difference was detected in the highest and lowest alarming call frequencies between groups (ANOVA: F_1_ = 17.34, df = 112, *P*_1_ < 0.01; F_2_ = 15.752, *P*_2_ = <0.01).

### Acoustic spectrum characteristics of mobbing calls

**Figure 2:**
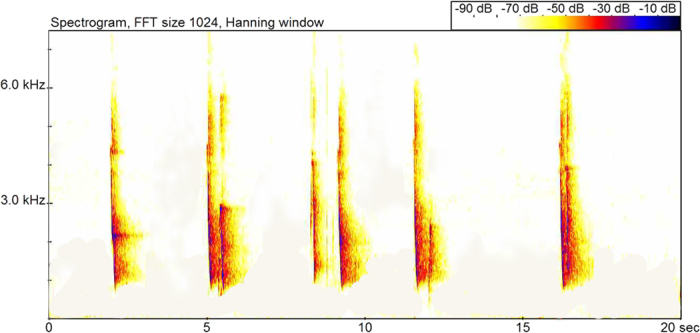
The mobbing call in a group of Hainan gibbons (group B).

Hainan gibbon groups emit a “jier-jier-jier” sound following the alarming call. The duration of the mobbing call was 5–9 min (Table 1) and was composed of long simple syllables of gradually decreasing frequency (wa notes; Fig. 2). There was a difference in the duration of mobbing call between groups (ANOVA: F = 4.33, df = 14, *P* < 0.05). The frequency of the mobbing call is higher than the alarming call. The mean duration of a mobbing call syllable was 0.154 ± 0.041 s at a frequency of 690–3920 Hz (Table 3). A difference was detected in the highest mobbing call frequency (ANOVA: F = 3.69, df = 70, *P* < 0.01) and lowest mobbing call frequency (F = 7.58, df = 70, *P* < 0.01) between groups.

### Responses to alarming calls

**Figure 3:**
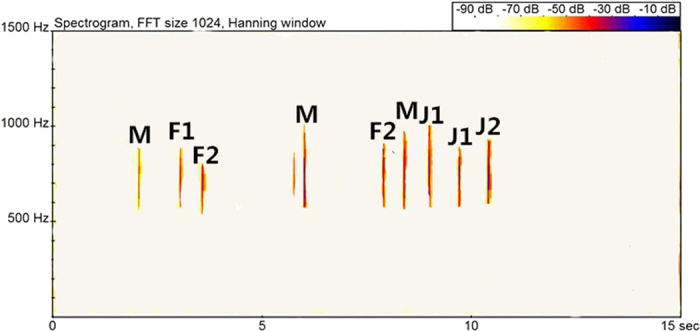
Individual responses to the alarming calls of Hainan gibbons (group C). Note: M represents an adult male, F represents an adult female and J represents a juvenile gibbon.

The response time of group members to the alarming call was 0.50 ± 0.38 s (Table 4). Individuals responded quickly to the alarming call, regardless of the distance between individuals in the family group (Fig. 3).

## Discussion

Spoken language is a result of the human capacity to assemble simple vocal units into more complex utterances, the basic carriers of semantic information. The vocal abilities of non-human primates are relatively unimpressive in comparison, with gibbon songs being a rare exception^9^. Acoustic signals play an important role in the responses of animals to external risks^14^, and alarm-calling behavior is an important component of nonhuman primate social communication^15^. The alarm calls of most animals are divided into functional and urgency reference systems according to the alarm functions of calls^16^,^17^. The alarm call in a functional reference system usually contains information about the type of natural enemy. When a species has more than one natural enemy, they may have a variety of alarm calls. The alarm call in the urgency reference system contains no information about the enemy type but contains information about urgency indicating the degree of danger perceived by the emitter. Hainan gibbons do not have different alarm calls for different threats, unlike vervet monkeys (*Cercopithecus aethiops*)^18^, ring-tailed lemurs (*Lemur catta*)^19^, Barbary monkeys (*Macaca sylvanus*)^20^,^21^, and Campbell’s monkey (*C. campbelli*)^22^. Further, Hainan gibbons did not adjust alarm call behavior according to different threats, as reported for *C. campbelli*^23^. The alarm calls of Hainan gibbons only contain simple short syllables (“aa note”) in the alarming call and longer variable-frequency syllables (“wa note”) in the mobbing call. In contrast, the simple alarming call in Hainan gibbons elicits a rapid response in group members and subsequent mobbing calls and behavior. This kind of alarm call only alerts other individuals in the group to the presence of an external threat, but does not help them identify the type of threat or what type of evasive strategy to use. Cotton-top tamarins (*Saguinus oedipus*) produce one type call when they see a dangerous animal, which usually causes animals to gather immediately; they use another type calls when they hear a danger signal, which makes individuals cease activity and look around^24^,^25^. The two types of alarm calls emitted by Hainan gibbons are similar with *S. oedipus*. Hainan gibbon alarm calls maybe a simple urgency reference system with limitations.

Cheney and Seyfarth^26^ and Fischer *et al.*^27^ posited that threats from predators in the sky and on the ground are key factors associated with primates that build a functional alarm system because they require different modes of escape. But Lar gibbon (*H. lar*) reliably sang in response to the terrestrial, but not the raptor, predator models, suggesting that singing is a firm part of these primates’ natural defense to ground predators^11^. During our field observations, Hainan gibbons made the same alarm call for all threats including in the sky and on the ground predators. Moreover, males and females >1.5 years old used the same alarm call, and this may be related to having few natural enemies in BNNR^28^.

The alarming behavior is the adaptation strategy of animals when they faced threaten from the habitat, especially for the primates and the group living animals. As for the Hainan gibbons’ alarming behavior when they encountered threatens from the ground, first they elicit alarming call, and then is the mobbing behavior, that means, the non-specific alarm calls trigger mobbing behavior. The alarming could remind the group members there were the dangerous, and the mobbing call could disturb the predators attentions, so as to protect themselves safety, when they emitted the mobbing call, Hainan gibbons just emitted the higher frequency vocal signals, not move or show any action, and never move together into one canopy, they always hide in the original canopy locations, just emitted the mobbing calls. Because there are not so many natural enemies in their habitat, there were not the big cats animals (such as *Neofelis nebulosa*) lived in Bawangling National Nature Reserve, the natural enemies of the Hainan gibbons, just some Hawk (*Spizaetus nipalensis* and *Ictinaetus malayensi*), and the human beings^28^. Mobbing can be silent, noisy, a physical attack or a combination of actions, but is not a reaction to a predator attack and is a response to a dangerous situation or presence. Mobbing, as any behavior related to predation, is an important force of natural selection^29^. The more interesting alarming behavior of the Hainan gibbons is the mobbing vocal behavior, and this behavior is first report among the primates’ species. The mobbing vocal behavior of Hainan gibbon is similar as birds and fishes, these animals use this behavior to disturb the predators then protect themselves. Mobbing calls by birds are effective^30^ and the alarming call behavior of Hainan gibbons which is similar to some avian alert behaviors; we think it is an effective way of defense strategy.

Alarm call of Hainan gibbon is simple, composed of only simple syllables and the frequency is low, but effective. This may be easier for young gibbons to learn, different gender and age stages of individuals have the two sound signals, and indicate that the Hainan gibbon is a simple primitive species in the family Hylobatidae.

## Methods

### Location and subjects

BNNR is located at the junction of Changjiang and Baisha counties in Hainan province (19°02′–19°08′N, 109°02′–109°13′E). The area is 300 km^2^ with an altitudinal range of 350–1438 m. Only three gibbon groups (group A: 1 male, 2 females, 2 sub-females, 2 juveniles, and 2 infants; group B: 1 male, 2 females, 2 juveniles and 1 infant; group C: 1 male, 2 females and 2 infants) and six solitary male gibbons remain in BNNR^13^. At the time of the study, large predators such as clouded leopard (*Neofelis nebulosa*) and black bear (*Selenarcto thibetanusform*) were absent from the reserve, but potential threats to Hainan gibbons, such as people, pythons and raptors remained^28^. We observed the responses of Hainan gibbons to these threats during field research.

### Data collection

All experimental protocols were approved by State Forest Administration. We not sample the specimens from the wild animals directly; just use the digital record equipment to record the vocal signals during the research times. The methods were carried out in “accordance” with the relevant guidelines, including any relevant details. We recorded calling behavior in the three groups and six solitary gibbons. Group B was tracked and observed in mainly during Sep 2002–Jan 2003, group A was observed in during Aug 2007–Dec 2007, and group C was observed in during Aug 2012–Jan 2013. These gibbons are familiar with researchers, but emit alarm calls in response to other people. We travelled to monitoring points before sunrise to hear the first morning calls during each field work day and then tracked the groups. Their behaviors were recorded when potential predators, such as snakes and raptors or human threats, appeared. A Samsung YV-150 recorder was used to record alarm calls of Hainan gibbons to threats (people, raptor, snake), and whole-event sampling was used to record the start and end times of alarm calls, song orientation, geographical coordinates and behavior. Each gibbon’s song had specific acoustic features, and individuals were easily distinguished from others via song, a feature of *Nomascus* species^31^. We identified response times in groups according to sound spectrum analysis and noted the individual order of participation during mobbing calls.

In total, we recorded 65 alarm call events over 129 d. We selected 15 alarm calls (every fifth event in groups A, B, and C) for analysis, none of which had background noise and were recorded within 30 m of each group.

### Acoustic spectrum analysis

Audio files were converted to WAV format, and Batsound 4.14 (Pettersson Elektronik AB, Uppsala, Sweden) sound analysis software was used to analyze the acoustic spectra of alarm calls^13^. Population differences, duration, response time, and highest and lowest frequency of the alarm call were analyzed using the following parameters: sampling frequency, 12 kHz; fast Fourier transform value for processing, 1,024; and Hamming analysis window length, 2,560. All song analysis and terms were according to Geissmann’s standards^6^.

### Statistical analysis

All data were normally distributed, and all results are expressed as mean ± standard deviation. One-factor analysis of variance (ANOVA) and the least significant difference (LSD) multiple comparisons test were used to test group differences in alarm call duration and the highest and lowest average frequency. We set the significance level to α = 0.05 and conducted all analyses using SPSS 18.0 (SPSS Inc., Chicago, IL, USA).

## Additional Information

**How to cite this article**: Deng, H. *et al.* Non-specific alarm calls trigger mobbing behavior in Hainan gibbons (*Nomascus hainanus*). *Sci. Rep.* **6**, 34471; doi: 10.1038/srep34471 (2016).

## Figures and Tables

**Table 1:** Duration of alarming and mobbing calls.

Family group	Alarming call duration time (min) Mean ± SD	Mobbing call duration time (min) Mean ± SD	Number
A	10.8 ± 1.30	7.6 ± 1.14	9
B	8.6 ± 1.52	6.2 ± 1.30	6
C	7.4 ± 1.14	5.4 ± 1.14	5

**Table 2:** Acoustic spectrum characteristics of alarming call made by Hainan gibbons.

Family group	Duration (s)	Mean value of the lowest frequency (Hz)	Mean value of the highest frequency (Hz)
A	0.078 ± 0.016	650 ± 40	870 ± 50
B	0.079 ± 0.012	670 ± 70	930 ± 66
C	0.079 ± 0.013	590 ± 60	850 ± 90
Mean	0.078 ± 0.014	640 ± 70	880 ± 80

**Table 3:** Acoustic spectrum characteristics of the mobbing call made by Hainan gibbons.

Family group	Duration (s)	Mean value of the lowest frequency (Hz)	Mean value of the highest frequency (Hz)
A	0.141 ± 0.047	980 ± 100	1970 ± 260
B	0.155 ± 0.044	1080 ± 170	2840 ± 440
C	0.167 ± 0.028	800 ± 60	3390 ± 360
Mean	0.154 ± 0.041	950 ± 180	2970 ± 600

**Table 4:** Response times of group members to alarming call.

Family group	Response time to alarming call	Shortest response time (s)	Longest response time (s)
A	0.74 ± 0.57	0.15	1.32
B	0.52 ± 0.39	0.06	0.94
C	0.34 ± 0.19	0.22	1.44
Mean	0.50 ± 0.38		
